# Association between diabetes-related factors and clinical periodontal parameters in type-2 diabetes mellitus

**DOI:** 10.1186/1472-6831-13-64

**Published:** 2013-11-07

**Authors:** Eun-Kyong Kim, Sang Gyu Lee, Youn-Hee Choi, Kyu-Chang Won, Jun Sung Moon, Anwar T Merchant, Hee-Kyung Lee

**Affiliations:** 1 Department of Preventive Dentistry, School of Dentistry, Kyungpook National University, Daegu, Republic of Korea; 2 Graduate School of Public Health, Yonsei University, Seoul, Republic of Korea; 3 Internal Medicine, College of Medicine, Yeungnam University, Daegu, Republic of Korea; 4 Department of Epidemiology and Biostatistics, Arnold School of Public Health, University of South Carolina, Columbia, SC, USA; 5 Department of Dentistry, College of Medicine, Yeungnam University Daegu, 317-1, Daemyung-dong, Nam-Gu, 705-717, Daegu, Republic of Korea

## Abstract

**Background:**

Evidence consistently shows that diabetes is a risk factor for increased prevalence of gingivitis and periodontitis. But there is a controversy about the relationship between diabetes related factors and periodontal health. The aim of the present study is to explore the relationship between diabetes related factors such as glycosylated hemoglobin, fasting blood glucose, duration of diabetes and compliance to diabetes self management and periodontal health status.

**Methods:**

Periodontal health of 125 participants with type-2 diabetes mellitus was measured by the number of missing teeth, community periodontal index (CPI), Russell’s periodontal index and papillary bleeding index. Information on sociodemographic factors, oral hygiene behavior, duration and compliance to self management of diabetes, levels of glycosylated hemoglobin(HbA1c) and fasting blood glucose(FBG) were collected by interview and hospital medical records. Statistically, independent t-test, an analysis of variance (ANOVA), chi-squared test and multiple regression analyses were used to assess the association between diabetes-related factors and periodontal health.

**Results:**

Periodontal parameters including the number of missing teeth and papillary bleeding index were significantly influenced by duration of diabetes, FBG and compliance to self management of diabetes. CPI was significantly influenced by duration of diabetes, FBG and HbA1C. And Russell’s periodontal index was significantly influenced by duration of diabetes, FBG, HbA1C and compliance to self management of diabetes. Results of multiple linear regression analysis showed that the duration of diabetes showed significant positive correlation with all of the periodontal health parameters, except for missing teeth. HbA1c was correlated with Russell's periodontal and papillary bleeding index. FBG and compliance to self management of diabetes were correlated with missing teeth and papillary bleeding index respectively.

**Conclusions:**

Diabetes-related factors such as duration of diabetes, FBG, HbA1c and compliance to self management of diabetes were significantly correlated with periodontal health among individuals with type-2 diabetes.

## Background

Periodontal disease, one of the most common chronic inflammatory diseases, with gradual destruction of connective tissue surrounding the teeth, eventually leads to tooth loss. Periodontal diseases include gingivitis (in which the inflammation is confined to the gingiva, and is reversible with good oral hygiene) and periodontitis (in which the inflammation extends and result in tissue destruction and alveolar bone resorption) [1]. Periodontitis occurs primarily in adults and its incidence increases with age [2]. According to a report published in 2010 on the oral health status of Koreans in Korea, 29.8% of participants in the 35-44-year-old category had a periodontal pocket greater than 4 mm, and this value increased to 55.2% in the 65-74-year-old category. In the 35-44-year-old category, only 21.8% of participants reported having healthy periodontium without gingival bleeding [3]. Periodontitis has negative impacts on many aspects of daily living and quality of life [4].

Among various risk factors for periodontitis, diabetes has been confirmed as a major risk factor [5,6]. The prevalence of periodontitis is higher and its symptoms are more severe in individuals with diabetes, compared with non-diabetics [5,7,8]. The influence of diabetes on oral health conditions has been well documented. Frequently observed oral conditions in patients with diabetes include dental caries, xerostomia (dry mouth), tooth loss, gingivitis, cheilitis, increase of glucose level in saliva, and periodontitis [9].

Diabetes mellitus (DM), resulting from a deficiency in insulin secretion or its action, is a metabolic disorder accompanying chronic complications such as micro vascular damage, nerve damage, and atherosclerosis [10]. Along with socioeconomic development and changes in lifestyle, the incidence of diabetes is increasing. According to a report published in 2009, 10.2% of males, 7.9% of females, and 9.1% of the total population in Korea have diabetes [11,12].

Many studies have reported on the correlation between various diabetes-related factors including HbA1c and duration of diabetes, and periodontal health. In the US National Health and Nutrition Examination Survey (NHANES) III, adults with an HbA1c level of >9% had a significantly higher prevalence of severe periodontitis than those without diabetes [13]. Most studies agree that HbA1c is associated with severity of periodontitis [14,15]. However, studies evaluating the relation between periodontitis and the duration of diabetes have conflicting results. For instance, Standberg and colleagues reported that duration of diabetes was not associated with periodontitis [16]. Other research groups have reported that the prevalence of periodontitis increased with the duration of diabetes [17-19].

In addition, the studies on association of periodontal health and diabetes behavior factor such as a healthy diet, physical exercising and self-monitoring of blood glucose, are rare. However, a report investigated that dental self-efficacy is associated with HbAlc level among diabetes patients [20]. By assessing compliance to self management of diabetes, which describes the degree to which a patient correctly follows medical advice about self management of diabetes such as diet, medication regimen and exercise, we tried to evaluate the relation between behavior factor of diabetes patient and periodontal health.

Therefore, we undertook this study to understand the association between various diabetes-related factors like duration of diabetes, HbA1c, fasting blood glucose and compliance to self management of diabetes and periodontal health.

## Methods

### Study participants

A total of 125 diabetic patients were recruited from the Department of Endocrinology at Yeungnam University Hospital, located in Daegu City, South Korea, from November 2005 to February 2006. Inclusion criteria were: (1) type-2 diabetes diagnosis by an endocrinologist for more than one year, (2) had 8 or more teeth and (3) did not have other general health problems such as cardiovascular, liver and kidney diseases, or other systemic conditions; including immunologic or psychiatric disorders. All participants provided written informed consent before enrollment. This research was approved by the Institutional Review Board at Yeungnam University (YUH-13-0395-O40).

### Oral examination and general information

A trained dentist performed an oral examination and examined dental panoramic X-ray, which provided information regarding Russell’s periodontal index [21]. For assessments of periodontal health, the number of missing teeth, Russell’s periodontal index, papillary bleeding index [22] and community periodontal index(CPI) [23] were measured using WHO-probe (Hu-Freidy, Chicago, IL, USA). Clinical criteria of each periodontal parameter were described in Table 1. Mean values of each periodontal parameter were calculated individually. A self-administrated questionnaire was provided to investigate the socio-demographic factors (age, gender and education), general health behavior (smoking and drinking), oral hygiene behaviors (toothbrush frequency and oral health education) and self-perceived oral health.

**Table 1:** Description of clinical diagnostic criteria for periodontal parameters

**Clinical diagnostic criteria**	**Description**
Community Periodontal index	Considers the worst condition encountered in six sites evaluated and used the following four codes:
	0 = healthy;
	1 = absence of pockets, bacterial plaque retention factors, or bleeding following probing;
	2 = depth as much as 3 mm and presence of bacterial plaque retention factors;
	3 = pockets with probing depth between 4 and 5 mm;
	4 = probing depth ≥ 6 mm
Russell’s periodontal index	The periodontal tissue of the remaining teeth including third molar were subjected and the stratification was the following.
	0: Negative. There is neither overt inflammation in the investing tissues nor loss of function due to destruction of supporting tissues.
	1: Mild gingivitis. There is an overt area of inflammation in the free gingival, but this area does not circumscribe the tooth.
	2: Gingivitis. Inflammation completely circumscribes the tooth, but there is no apparent break in the epithelial attachment.
	4: There is early, notch like resorption of the alveolar crest.
	6: There is horizontal bone loss involving the entire alveolar crest, up to half of the length of the tooth root (distance from apex to cemento-enamel junction).
	8: There is advanced bone loss, involving more than one-half of the length of the tooth root; or a definite intrabony pocket with definite widening of the periodontal ligament. There may be root resorption, or rarefaction at the apex.
Papillary bleeding index	The interdental sites were probed in order from the right maxillary second molar to the left maxillary second molar (#17, 16, 11, 26 & 27) and from the left mandibular second molar to the right mandibular second molar (#37, 36, 31, 46 & 47). the stratification was the following.Score 0-no bleeding;
	Score 1-A single discreet bleeding point;
	Score 2-Several isolated bleeding points or a single line of blood appears;
	Score 3-The interdental triangle fills with blood shortly after probing;
	Score 4-Profuse bleeding occurs after probing; blood flows immediately into the marginal sulcus.

### Diabetes-related factors

**Figure 1:**
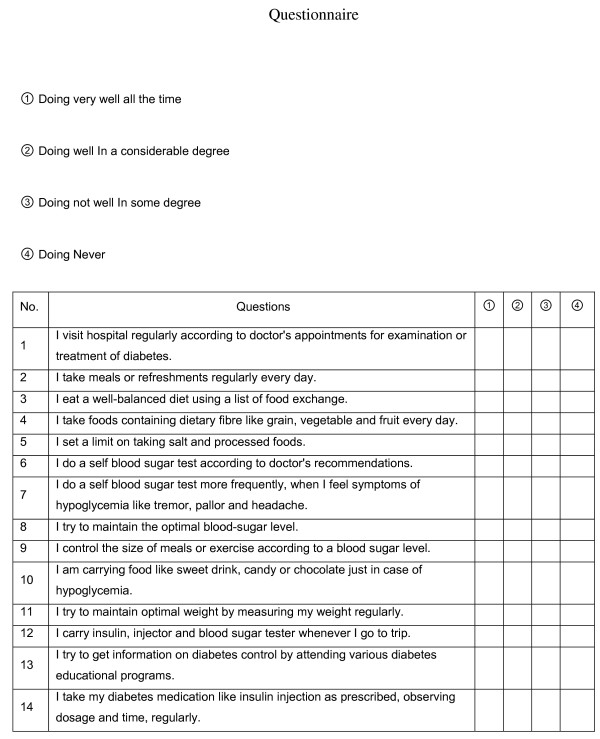
Questionnaire on compliance to diabetes self management.

Information on HbA1c, expressed as the percentage of hemoglobin that is glycosylated and FBG were collected from the medical records of participants who had visited hospital regularly, with their permission. Other diabetes-related factors, including duration and compliance to self management of diabetes were obtained by questionnaire by the dental hygienist. Questionnaire on compliance to diabetes self management, modified from the questionnaire developed by Park (1985) consists of 14 questions (4 questions about diet control, 2 about exercise, 2 about insulin treatment, 5 about self-monitoring blood glucose and general management, and 1 about diabetic education) [24,25] (Figure 1). Each question was rated on a 4-point Likert scale (1 = all of the time and 4 = never) with higher scores indicating poor diabetes management. Mean value of all question was calculated individually.

### Statistical analysis

Comparisons of four periodontal health parameters by diabetes related factors were performed by independent t-test, one-way ANOVA and chi-squared test. Multiple regression analyses were adopted for exploration of the explainable variables for periodontal health parameters by sociodemographic factors, diabetes-related factors and oral health behaviors. All statistical analyses were implemented using the statistical analysis software of SPSS (SPSS 19.0 for Windows, SPSS Inc, USA). Statistical significance level was set at 0.05.

## Results

1 Distribution of socio-demographic characteristics, smoking, drinking and oral hygienic behaviors

The distribution of the socio-demographic, lifestyle and oral hygiene behavior factors across periodontal parameters was shown in Table 2. The mean age of participants was 57.8 years (range: 33-75 years) and 61.6% of participants were male. The education level of the majority of subjects was below middle school graduation (57.6%). The prevalence of smoking and drinking was 24.0% and 40.8%, respectively. The most reported frequency of tooth brushing was two times per day (56.8%). Only 20.0% of subjects reported having oral health education. For the question asked about self-perception on oral health status, majority of subjects reported “unhealthy” (72.8%).

2 Periodontal health status by diabetes-related factors

The distribution of the diabetes-related factors across periodontal parameters was shown in Tables 3 and 4. The duration of diabetes and FBG of the majority of subjects was 6 to 9 years (59.2%) and 140 to199 mg (40.0%), respectively. HbA1c level above 7% was observed in 69.6% of subjects. The compliance to self management of diabetes was classified into two categories (Do-well: participants whose score of self management of diabetes > mean value (2.40) of compliance to self management of diabetes score of all participants, Not do–well: the others): 56.0% of subjects belonged to the Do-well category. The number of missing teeth and score of papillary bleeding index increased significantly in the categories of longer duration of diabetes, higher FBG level and Not-do-well of compliance to self management of diabetes. Participants with longer duration of diabetes, higher FBG level and HbA1C ≥7 showed significantly higher code of CPI and Russell’s periodontal index which means poorer periodontal health. Participants in Not-do-well category of compliance to self management of diabetes variable showed significantly higher code of Russell’s periodontal index.

3 Multiple regression analysis

In multiple linear regression analysis, duration of diabetes significantly predicted all periodontal health parameters, except for missing teeth. Missing teeth was significantly correlated with age and FBG (*R*^*2*^ = 0.535, *p* < 0.001). CPI was significantly correlated with smoking, education level, duration of diabetes and oral health education (R^2^ = 0.400, *p* < 0.001). Russell’ periodontal index was significantly correlated with duration of diabetes, HbA1c and oral health education (R^2^ = 0.542, *p* < 0.001). And papillary bleeding index was significantly correlated with education level, duration of diabetes, HbA1c and compliance to self management of diabetes (R^2^ = 0.433, *p* < 0.001) (Table 5).

**Table 2:** Distribution of socio-demographic, smoking, drinking and oral hygiene behavior

**Variables**	**N**	**%**
Gender		
Male	77	61.6
Female	48	38.4
Age (years)		
30-39	10	8.0
40-49	25	20.0
50-59	28	22.4
60-69	40	32.0
70-	22	17.6
Mean ± S.E	57.85 ± 1.03	
Smoking		
Yes	30	24.0
No	95	76.0
Drinking		
Yes	51	40.8
No	74	59.2
Education level		
≤Middle school	72	57.6
High school	34	27.2
College≤	19	15.2
Frequency of tooth brush (per day)		
1 times	13	10.4
2 times	71	56.8
3 times	41	32.8
Oral health education		
Yes	25	20.0
No	100	80.0
Perceived self-oral health status		
Healthy	34	27.2
Unhealthy	91	72.8

**Table 3:** Distribution of missing teeth and papillary bleeding index by diabetes-related factors

**Variables**	**No (%)**	**Missing teeth**	***p***	**Papillary bleeding index**	***p***
		**Mean ± S.E**		**Mean ± S.E**	
Duration of diabetes illness					
≤5 years	36 (28.8)	3.14 ± 0.60	0.000	1.90 ± 0.11	0.000
6-9 years	74 (59.2)	4.96 ± 0.41		2.09 ± 0.08	
≥10 years	15 (12.0)	8.40 ± 1.44		2.75 ± 0.15	
Fasting blood glucose (mg/dl)					
<140	26 (20.8)	2.15 ± 0.39	0.000	1.89 ± 0.10	0.014
140-199	50 (40.0)	3.72 ± 0.50		2.02 ± 0.11	
≥200	49 (39.2)	7.43 ± 0.59		2.34 ± 0.09	
HbA1c (%)					
<7	38 (30.4)	3.79 ± 0.58	0.057	1.97 ± 0.12	0.123
≥7	87 (69.6)	5.31 ± 0.46		2.11 ± 0.06	
Compliance to self management of diabetes					
Do-well	70(56.0)	420 ± 0.46	0.046	1.91 ± 0.08	<0.001
Not do-well	55(44.0)	5.67 ± 0.58		2.39 ± 0.08	

*p* by t-test or one-way ANOVA.

**Table 4:** Distribution of CPI and Russell’s periodontal index by diabetes-related factors

**Variables**	**N (%)**	**CPI**				**Russell’s periodontal index**			
		**Code 0,1,2**	**Code 3**	**Code 4**	***p***	**Code 0,1,2,4**	**Code 6**	**Code 8**	***p***
		**N(%)**	**N(%)**	**N(%)**		**N(%)**	**N(%)**	**N(%)**	
Duration of diabetes illness									
≤5 years	36 (28.8)	5(13.9)	17(47.2)	14(38.9)	0.018	5(13.9)	14(38.9)	17(47.2)	0.036
6-9 years	74 (59.2)	2(2.7)	33(44.6)	39(52.7)		3(4.1)	30(40.5)	41(55.4)	
>10 years	15 (12.0)	0(0.0)	3(20.0)	12(80.0)		0(0.)	2(13.3)	13(86.7)	
Fasting blood glucose (mg/dl)									
<140	26 (20.8)	5(19.2)	16(61.5)	5(19.2)	<0.001	2(7.7)	12(46.2)	12(46.2)	0.007
140-199	50 (40.0)	2(4.0)	22(44.0)	26(52.0)		6(12.0)	22(44.0)	22(44.0)	
>199	49 (39.2)	0(0.0)	15(30.6)	34(69.4)		0(0.0)	12(24.5)	37(75.5)	
HbA1c (%)									
<7	38 (30.4)	5(13.2)	19(50.0)	14(36.8)	0.013	6(15.8)	19(50.0)	13(34.2)	0.001
≥7	(69.6)	2(2.3)	34(39.1)	51(58.6)		2(2.3)	27(31.0)	58(66.7)	
Compliance to self management of diabetes									
Do-well	70(56.0)	6(8.6)	32(45.7)	32(45.7)	0.127	8(11.4)	27(38.6)	35(50.0)	0.021
Not do-well	55(44.0)	1(1.8)	21(38.2)	33(60.0)		0(0.0)	19(34.5)	36(65.5)	

*p* by chi-squared tests.

**Table 5:** Multiple linear regression analysis for Missing Teeth, CPI, Russell’s periodontal index, and Papillary bleeding index

**Variables**	**Missing teeth**	**CPI**	**Russell’s periodontal index**	**Papillary bleeding index**
	Parameter estimate* (Standard error), *P-* **value**			
Gender (ref. female)				
Age	.185 (.029) < .001			
Smoking (ref. No)		-.375 (.139) .008		
Drinking (ref. No)				
Education level (ref. ≤Middle school)				
High school vs. ≤Middle school				-.415(.142) .004
College ≤ vs. ≤Middle school		-.478 (.181) .010		-.496(.176) .006
Duration of diabetes illness (ref. ≤5 years)				
6-9 years vs. ≤5 years			.513 (.223) .023	
>10 years vs. ≤5 years		.461 (.204) .026	1.306(0.353) < .001	.677(.199) < .001
Fasting blood glucose (mg/dl)	.022(.006) < .001			
HbA1c (%)			.121 (.060) .045	.072 (.034) .036
Compliance to self management of diabetes (ref. Not-do-well)				-.339 (.122) .007
Frequency of tooth brush (per day)				
Oral health education (ref. No)		-.313 (.157).048	−1.789 (.270) < .001	
Perceived self-oral health status (ref. Unhealthy)				
	R^2^ = .535, *p <* .001	R^2^ = .400, *p <* .001	R^2^ = .542, *p <* .001	R^2^ = .433, *p <* .001

*Adjusted for gender, age, smoking, drinking, education level, frequency of tooth brush, oral health education and perceived self-oral health status.

## Discussion

The periodontal health of this group of individuals with type-2 diabetes was poorer with increased duration of diabetes, higher levels of FBG, HbA1c and higher score of compliance to self management of diabetes. Longer diabetes duration, higher FBG and HbA1C levels, and poorer score of compliance to self management of diabetes were positively related to poorer scores on periodontal health parameters in multiple linear regression analysis.

As mentioned in the introduction, studies evaluating the relationship between duration of diabetes and periodontitis have inconsistent results [16-19]. In this study, all periodontal parameters including missing teeth, CPI, Russell’s periodontal index and papillary bleeding index were significantly related to duration of diabetes. Also, compliance to self management of diabetes showed significant relationship with all periodontal parameter except for CPI. Generally, results of cross-sectional study cannot be interpreted as a causal relationship. But as reported by Kneckt MC, participants who have good compliance to self management of diabetes tend to have higher dental self-efficacy which is related to good periodontal health [26]. On the other hand, good compliance to self management of diabetes may result in well control of HbA1c, which is reported to be associated to periodontal health. Therefore in light of these, we think that our result is plausible. FBG and HbA1C levels showed significant relationship with periodontal parameters, which are coincident with preceding studies [14,15].

For comprehensive evaluation of periodontal health, we measured several periodontal indices (CPI, Russell’s periodontal index and papillary bleeding index). CPI is a widely used method to assess and summarize periodontal health status in epidemiological studies but is sensitive to individual error when measuring periodontal pocket depth [27]. To overcome this limitation we used the Russell’s periodontal index, with dental panoramic x-ray to assess periodontal health objectively. In panoramic X-ray, interproximal alveolar bone loss can be detected easily, but alveolar bone loss occurring on buccal, lingual or palatal sites cannot be detected. We therefore combined the two methods (CPI, Russell’s periodontal index) to assess periodontal health more accurately, together with papillary bleeding index to assess gingivitis. Strength of this study was the comprehensive assessment of periodontal health using these three complementary indices.

However, this study had several limitations. First, we did not measure the intra-examiner error which is important to clinical research with CPI, to alleviate participant’s sufferings. Second, we investigated the current state of smoking in questionnaires, so ex-smokers might have been classified as non-smoker group in questionnaires. Periodontal health of ex-smokers might have been affected by past smoking experience and this result might have influenced on the result of multiple regression analysis which was adjusted for smoking variables. Therefore, further studies need to evaluate smoking status of participants considering the number of cigarettes, experience and period of smoking.

Periodontitis is reversible in patients with well-controlled diabetes and the outcome of its treatment in patients with diabetes is similar to that in non-diabetics [28]. In addition, periodontitis is preventable with adequate oral hygiene practice [29]. In this study, oral health education has been shown to have a significant influence on periodontal health among patients with type-2 diabetes mellitus. Diabetes is a chronic disease requiring lifelong treatment, and patients with a long duration of diabetes have demonstrated a propensity to neglect their oral health [30]. So, it is desirable that diabetes health care professional and dentist promote diabetes self-care as well as oral health education for management of both diabetes and periodontal health, especially among patients with long duration of diabetes.

## Conclusions

Diabetes-related factors such as duration of diabetes, FBG, HbA1c and compliance to self management of diabetes were significantly correlated with periodontal health among individuals with type-2 diabetes. Therefore, we emphasize the role of blood glucose control and self-care practice of diabetes along with oral health education for management of oral health, which is exceptionally important for prevention of diabetes related complications and improvement of quality of life [31,32].

## Competing interests

The authors declare that they have no competing interests.

## Authors' contributions

E-KK and H-KL designed the study, performed data analyses, and drafted the original paper. Y-HC trained and supervised dentist and dental hygienist to collect data. K-CW transferred patients to dental clinic. J-SM provided clinic data related to diabetes. S-GL and ATM provided comments on the original draft and contributed to the development of the final draft. All authors read and approved the final manuscript.

## Pre-publication history

The pre-publication history for this paper can be accessed here:

http://www.biomedcentral.com/1472-6831/13/64/prepub
